# Beverage patterns and trends among school-aged children in the US, 1989-2008

**DOI:** 10.1186/1475-2891-10-103

**Published:** 2011-10-02

**Authors:** Gentry Lasater, Carmen Piernas, Barry M Popkin

**Affiliations:** 1 Department of Nutrition, Gillings School of Global Public Health, University of North Carolina at Chapel Hill, Chapel Hill, North Carolina, USA

**Keywords:** sugar-sweetened beverages, milk, diet drinks, children

## Abstract

**Background:**

High intake of sugar-sweetened beverages in childhood is linked to increased risk of obesity and type II diabetes later in life. Using three nationally representative surveys of dietary intake, we investigated beverage patterns and trends among US school-aged children from 1989/91 to 2007/08.

**Methods:**

3, 583 participants ages 6-11 y old were included. We reported per capita trends in beverage consumption, percent consuming, and amount per consumer for the following categories of beverages: sugar-sweetened beverages (SSB), caloric nutritional beverages (CNB) and low calorie beverages (LCB). Statistically significant differences were tested using the Student's t test in Stata 11.

**Results:**

While per capita kcal contribution from total beverages remained constant over the study period, per capita consumption of SSBs increased and CNBs decreased in similar magnitude. The substantial increase in consumption of certain SSBs, such as fruit drinks and soda, high fat high sugar milk, and sports drinks, coupled with the decrease in consumption of high fat low sugar milk was responsible for this shift. The percent consuming SSBs as well as the amount per consumer increased significantly over time. Per capita intake of total milk declined, but the caloric contribution from high fat high sugar milk increased substantially. Among ethnicities, important differences in consumption trends of certain SSBs and 100% juice indicate the complexity in determining strategies for children's beverage calorie reduction.

**Conclusions:**

As upward trends of SSB consumption parallel increases in childhood obesity, educational and policy interventions should be considered.

## Background

Extensive attention has focused on improving diet quality and reducing the total caloric intake of US children's diets as a way to both prevent further increases in obesity and to improve child health. Sugar-sweetened beverages have received particular attention among the pediatrics profession as have other caloric beverages such as 100% fruit juices and fruit drinks [1]. These and other foods with excessive amounts of added sugar and saturated fat represent a growing component of the diet of US children [2].

Many risks are associated with high intake of sugar-sweetened beverages in childhood. Previous studies have shown significant, positive associations between SSB consumption and weight gain among children [3,4]. Other studies have provided evidence that children consuming these beverages may have increased risk for the developing type II diabetes, metabolic syndrome, and obesity later in life [5,6]. These risks can be attributed to the body's low satiety response to liquid calories and a poor ability to compensate for these calories by reducing caloric intake in other areas, leading to potential weight gain over time [5,6]. Beverages with high sugar content also increase the risk of developing dental caries, which is of high concern for this age group [7].

A number of studies have highlighted increased consumption of an array of unhealthy, high sugar beverages over the past two decades among American children, along with decreased intake of milk [4,8,9]. Several earlier studies have focused attention on 6-11 y olds [10-12]. Another suggested that fruit drinks are becoming just as important for 2 to 11 year olds, finding that soft drinks contributed to less than half of the total SSB consumption [8]. Different research from our team found increased consumption of high fat sugar-sweetened milks in some European nations where the schools have banned sugar-sweetened beverages [13,14].

In the US and a large number of other countries, efforts have been made to reduce intake of all carbonated sugar-sweetened beverages and all fruit drinks. The American Academy of Pediatrics, recommends in its last policy statement that sugar-sweetened beverages and naturally sweet beverages, such as fruit juice, should be limited to 4 to 6 oz per day for children 1 to 6 years old, and to 8 to 12 oz per day for children 7 to 18 years old [15,16]. In many situations, global beverage associations have attempted to replace these items with either 100% fruit juice or sugar-sweetened milk, usually whole or high fat milk. The American Beverage Association with the support of the Clinton Foundation and the American Heart Association proposed different guidelines in 2006 [17]. A major component of that agreement was to cap the number of calories in beverages in schools at 100 calories per container with milks and juices being excluded. This allowed the beverage companies to shift the beverage mix to sports drinks, juice and sugar-sweetened whole milk [18,19]. This foundation later released a report that stated of the 2009-2010 school year, 98.8% of all measured schools were in compliance [20].

The present study highlights trends in SSB consumption in the period before and during the implementation of this new approach to SSB promotion. We present updated patterns and trends in beverage consumption among US school-aged children ages 6 to 11. Using three nationally representative surveys of food intake in the US, from 1989/91 to 2007/08, we report per capita trends in beverage consumption, percent consuming, and amount per consumer for the following categories of beverages: sugar-sweetened beverages (SSB), such as sodas and sports drinks, caloric nutritional beverages (CNB), such as 100% fruit juice, and low calorie beverages (LCB), such as diet drinks and skim milk. To further understand differential patterns and trends among the studied subjects, we studied the same trends across different ethnic categories.

## Methods

### Survey Design and Sample

Participants were 3, 583 children aged 6-11 y old who participated in three US nationally representative dietary recall surveys: 1, 525 participants from the 1989-1991 Continuing Survey of Food Intake by Individuals (CSFII 89) [21]; 977 participants from the 2005-2006 National Health and Nutrition Examination Survey (NHANES 05-06) [22]; and 1081 participants from the 2007-2008 National Health and Nutrition Examination Survey (NHANES 07-08) [23]. All three surveys were designed to be nationally representative and are based on a multistage and stratified area probability sample of non institutionalized U.S. households. More detailed information for each survey may be found elsewhere [21-23].

Comparing data across the three surveys must take into account changes in survey methodology and operations over time. Since its integration with USDA's CSFII in 2002, NHANES methodology (sampling design, food composition tables and dietary collection methods) has been based on that of earlier CSFII surveys [24].

This study was determined to be exempt from institutional review board concerns since it utilized publicly available USDA and NHANES data.

### Dietary Records

In the CSFII89 survey, dietary intake was collected over three consecutive days using single interviewer-administered 24-hour dietary recall and two days of self-administered food record. The main meal planner/preparer was asked to report intake information for any children under the age of 12. Dietary intake for NHANES05-06 and NHANES 07-08 was based on 24-h dietary recall data from two nonconsecutive days (day one interviews were in-person interviews conducted at the Mobile Exam Center, while day two interviews were conducted by telephone from a central NHANES telephone center). For children under the age of 16, interviews were conducted with a proxy. To ensure comparability and consistency with the later surveys, the first two days of intake from the CSFII survey have been included in this study.

### Beverages Grouping System and Category Definitions

The grouping system developed by our research group summarizes intakes of beverages in a nutritionally meaningful way. Our beverage grouping system starts with the major USDA beverages groupings and systematically disaggregates them into 21 independent categories of beverages. Cutoff points for sugar and fat were applied to separate the different categories of milk. Since the 2010 Dietary Guidelines for Americans promote intake of skim or low fat (1%) milk, we categorized milk beverages containing up to 1% fat as "low fat" whereas those containing more than 1% of fat were considered as "high fat" [25]. To determine a meaningful cut-off point for sugar content in milks, we calculated the average amount of intrinsic sugar in all plain milks and found that it was 6%. We considered beverages containing more than 6% of total sugar as "high sugar" and those lower than 6% as "low sugar" [26]. The beverages studied in this paper included coffee (unsweetened, diet and sweetened with sugar/milk), tea (unsweetened, diet and sweetened with sugar/milk), soft drinks (sugar and diet), fruit drinks (sugar and diet), sports drinks, 100% fruit/vegetable juice, milk and milk drinks, and other drinks (sugar and diet). Water was excluded due to data collection differences among the surveys.

Three categories of beverages were determined for study purposes. Sugar-sweetened beverages (SSBs) included high calorie beverages with little nutritional content, such as soft drinks and fruit drinks, high/low fat high sugar milk, sport drinks, sweetened tea/coffee and other sugar drinks. Caloric-nutritional beverages (CNBs) included caloric beverages with some nutritional benefits, such as high fat low sugar milk and 100% fruit/vegetable juice. Healthier beverages were those beverages low in calories and/or with nutritional benefits, such as low fat low sugar milk, unsweetened and diet tea/coffee, and diet drinks.

### Statistical Analysis

Data are presented as means ± SE. STATA 11 was used to perform all the statistical analysis [27]. Survey commands (SVY: MEAN) were used to account for survey design and weighting. For each year surveyed, trends on beverages are reported as mean kcal per day per capita, mean kcal per day per consumer and percent of individuals consuming for 3 categories of beverages: sugar-sweetened beverages (SSB), caloric nutritional beverages (CNB) and low calorie beverages (LCB). Similar trends were studied for selected categories of SSB and CNB across three categories of self-reported race/ethnicity, Hispanic, non-Hispanic White and non-Hispanic Black, and income. To more accurately represent income level, household income is expressed as a percentage of the federal poverty thresholds: low income was defined as less than 130%, medium as greater than or equal to 130% and less than 300%, and high as greater than or equal to 300%. Poverty thresholds are provided by USDA surveys to reflect the eligibility cut-offs for School Feeding Program and Supplemental Nutrition Assistance Program (SNAP) [21-23]. Statistically significant differences were tested using the Student's t test in Stata 11. A two sided P value of 0.05 was set to denote statistical significance.

## Results

### Demographic characteristics

Table 1 summarizes the sociodemographic characteristics of included children aged 6 to 11 from the three survey periods (CSFII89, NHANES05-06, and NHANES 07-08).

**Table 1:** Demographic Characteristics^1^.

	CSFII 1989-1991	NHANES 2005-2006	NHANES 2007-2008
Number of Observations	1171	977	1081
Gender (%)			
Male	52	50	51
Female	48	50	49
Race/Ethnicity (%)			
Hispanic	8	16	21
White	70	60	58
Black	16	14	15
Other	6	10	6
Income Level (%)			
Low	25	26	32
Medium	39	30	31
High	36	44	37
Maternal Education (%)			
< = High School	54	43	48
> High School	46	57	52

^1 ^The studied sample included children aged 6-11 y old from 3 nationally representative surveys: CSFII 1989-1991, NHANES 2005-2006 and 2007-2008.

^2^Income level was based on percent poverty threshold level: Low income was defined as less than 130%, medium as greater than or equal to 130% and less than 300%, and high as greater than or equal to 300%

### Recent trends for major beverage categories

**Figure 1:**
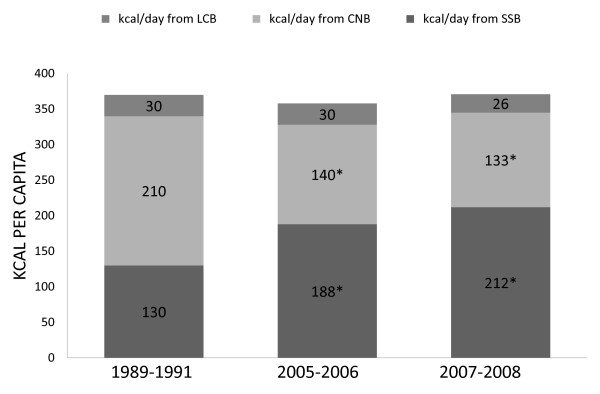
**Recent trends (kcal per capita) in beverage categories in US children 6-11 y old, 1989-2008, nationally representative^1, 2^**. ^1 ^The studied sample included children aged 6-11 y old from 3 nationally representative surveys: CSFII 1989-1991, NHANES 2005-2006 and 2007-2008. ^2 ^Differences were tested using Student's t test. Comparing with 1989: * P < 0.05, comparing with 2005: † P < 0.05.^3 ^Beverage category definitions: Low calorie beverages (LCB), caloric nutritional beverages (CNB), sugar-sweetened beverages (SSB)

Figure 1 shows trends in beverage consumption from the three major beverage categories: sugar-sweetened beverages (SSBs), caloric nutritional beverages (CNBs), and healthier beverages (Figure 1). From 1989-2008, per capita total caloric contribution from beverages remained constant, while the consumption of major types of beverages changed significantly. Important shifts in per capita trends of SSBs and CNBs occurred during the studied period. Total per capita calories from SSBs increased significantly from 1989 to 2008 (130 to 212 kcal/d, P < 0.05), while those from CNBs decreased in similar magnitude and significance over the same time period (210 to 133 Kcal/d, P < 0.05). Per capita caloric intake of healthier beverages did not change from 1989 to 2008. Short-term trends, from 2005-2008, did not show any significant change.

Several key beverage groups were responsible for the broad shifts in SSB and CNB consumption from 1989 to 2008. Table 2 shows the per capita, percent consuming, and amount per consumer trends for major beverage types of each category (Table 2). Within the SSBs, those that showed the largest increase in per capita trends were fruit drinks and soft drinks (90 to 118 kcal/d, P < 0.05), high fat high sugar milk (28 to 63 kcal/d, P < 0.05), and sports drinks (1 to 9 kcal/d, P < 0.05). In addition, beverage trends in mL/d gave insight into how beverage portion sizes are changing (Table 3).

**Table 2:** Per capita trends (kcal/d), amount per consumer, % consumers by beverage category in US children 6-11 y old (1989-2008), nationally representative^1, 2^.

	CSFII 1989-1991					NHANES 2005-2006					NHANES 2007-2008				
	Per Capita Trends (kcal/day)		**% Cons**.	Amt. Per Cons. (kcal/day)		Per Capita Trends (kcal/day		**% Cons**.	Amt. Per Cons. (kcal/day)		Per Capita Trends (kcal/day)		**% Cons**.	Amt. Per Cons. (kcal/day)	
**Sugar Sweetened Bev. (SSB)**	**130**	**± 11**	**79%**	**166**	**± 11**	190*	**± 9**	88%*	**214**	**± 8**	209*	**± 8**	91%*	**231**	**± 9**
fruit drinks and soda	90	± 6	67%	134	± 6	114*	± 6	79%*	144	± 6	118*	± 6	77%*	153	± 7
low fat high sugar milk	1	± 1	2%	88	± 9	15*	± 2	12%*	116	± 9	5*†	± 1	6%*†	88	± 6
high fat high sugar milk	28	± 5	19%	147	± 12	44*	± 4	28%*	160	± 7	63*†	± 4	39%*†	163	± 9
sports drinks	1	± 0	2%	64	± 9	9*	± 1	11%*	84	± 11	9*	± 1	12%*	76	± 10
sweetened tea and coffee	10	± 2	13%	73	± 8	7	± 1	12%	59	± 6	12 V	± 2	19%†	63	± 6
other sugar drinks	0	± 0	1%	24	± 8	1	± 0	1%	84	± 26	2*	± 1	2%	126	± 32
**Caloric Nutritional Bev. (CNB)**	**210**	**± 4**	**92%**	**228**	**± 5**	140*	**± 5**	83%*	**170**	**± 6**	134*	**± 6**	81%*	**166**	**± 6**
high fat low sugar milk	168	± 4	84%	200	± 7	97*	± 4	67%*	146	± 5	85*	± 5	64%*	134	± 6
100% juice (fruit + vegetable)	42	± 2	46%	91	± 5	43	± 3	49%	88	± 3	48	± 5	50%	97	± 6
**Low Calorie Bev. (LCB)**	**30**	**± 2**	**33%**	**91**	**± 8**	**30**	**± 5**	42%*	**72**	**± 9**	**27**	**± 3**	**40%**	**66**	**± 5**
low fat low sugar milk	21	± 1	15%	142	± 14	24	± 4	23%*	104	± 10	17	± 2	19%	90	± 7
unsweetened tea and coffee	0	± 0	5%	2	± 0	0	± 0	2%*	3	± 1	0	± 0	3%	2	± 0
total diet drinks	9	± 2	17%	54	± 6	6	± 1	24%	25	± 3	10†	± 2	26%*	38	± 4
diet drinks	0	± 0	5%	3	± 1	1*	± 0	15%*	6	± 1	1*	± 0	13%*	9	± 1
diet tea and coffee	9	± 2	12%	73	± 10	5	± 1	10%	52	± 7	9†	± 1	15%†	58	± 7

^1 ^The studied sample included children aged 6-11 y old from 3 nationally representative surveys: CSFII 1989-1991, NHANES 2005-2006 and 2007-2008.

^2 ^Differences were tested using Student's t test. Comparing with 1989: * P < 0.05, comparing with 2005: † P < 0.05.

**Table 3:** Per capita trends (mL/day), Amount per consumer (mL/day), % Consumers. Children aged 6-11 y old. US Nationally Representative, 1989-2008^1^.

	CSFII 1989-1991					NHANES 2005-2006					NHANES 2007-2008				
	Per Capita Trends (mL/day)		**% Cons**.	Amt. Per Cons. (mL/day)		Per Capita Trends (mL/day)		**% Cons**.	Amt. Per Cons. (mL/day)		Per Capita Trends (mL/day)		**% Cons**.	Amt. Per Consumer (mL/day)	
**Sugar Sweetened Bev. (SSB)**	**304**	**± 22**	**80%**	**381**	**± 18**	418*	**± 21**	88%*	**473**	**± 19**	468*	**± 21**	91%*	**517**	**± 23**
fruit drinks and soda	226	± 13	69%	330	± 12	279*	± 16	79%*	351	± 17	292*	± 16	77%*	377	± 17
low fat high sugar milk	3	± 1	2%	164	± 17	24*	± 4	12%*	193	± 14	8*†	± 1	6%*†	151	± 10
high fat high sugar milk	35	± 7	19%	183	± 17	53*	± 6	28%*	193	± 8	78*†	± 6	39%*†	203	± 12
sports drinks	5	± 0	2%	255	± 36	34*	± 5	11%*	317	± 40	36*	± 4	12%*	289	± 37
sweetened tea and coffee	33	± 6	13%	247	± 16	27	± 4	12%	224	± 23	49†	± 7	19%†	264	± 24
other sugar drinks	1	± 1	1%	172	± 79	1	± 1	1%	116	± 34	4	± 1	2%	241	± 38
**Caloric Nutritional Bev. (CNB)**	**389**	**± 9**	**92%**	**423**	**± 10**	273*	**± 10**	83%*	**331**	**± 11**	260*	**± 12**	81%*	**322**	**± 12**
high fat low sugar milk	299	± 10	84%	357	± 13	182*	± 8	67%*	273	± 9	160*	± 10	64%*	251	± 10
100% juice (fruit + vegetable)	90	± 5	46%	194	± 12	91	± 6	49%	186	± 6	100	± 10	50%	202	± 13
**Low Calorie Bev. (LCB)**	**102**	**± 7**	**33%**	**306**	**± 21**	**126**	**± 18**	44%*	**286**	**± 29**	**125**	**± 10**	43%*	**290**	**± 14**
low fat low sugar milk	53	± 2	15%	358	± 34	62	± 11	23%*	265	± 25	43	± 6	19%	229	± 20
unsweetened tea and coffee	9	± 2	5%	157	± 17	3*	± 1	2%*	192	± 64	5	± 1	3%	154	± 34
total diet drinks	41	± 7	17%	235	± 14	61	± 9	26%*	233	± 24	78*	± 7	29%*	267	± 16
diet drinks	10	± 2	5%	197	± 3	38*	± 7	17%*	225	± 25	36*	± 4	16%*	219	± 18
diet tea and coffee	31	± 6	12%	247	± 22	22	± 4	10%	228	± 28	42†	± 6	15%†	277	± 28

^1 ^The studied sample included children aged 6-11 y old from 3 nationally representative surveys: CSFII 1989-1991, NHANES 2005-2006 and 2007-2008.

^2 ^Differences were tested using Student's t test. Comparing with 1989: * P < 0.05, comparing with 2005: † P < 0.05.

From 2005-2008, per capita caloric consumption of high fat high sugar milk notably increased (from 44 to 63 kcal/d, P < 0.05). Total per capita calories from high fat low sugar milk contributed to the decrease in CNB per capita trends (from 168 to 86 kcal/d, P < 0.05).

**Figure 2:**
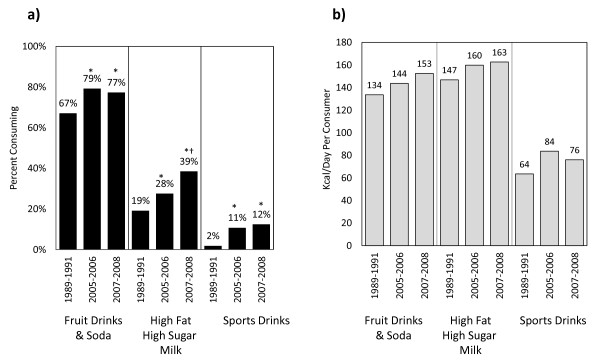
**a) Percent consuming and b) Amount per consumer (kcal/d) from beverage categories in US children 6-11 y old (1989-2008), nationally representative^1, 2^**. ^1 ^The studied sample included children aged 6-11 y old from 3 nationally representative surveys: CSFII 1989-1991, NHANES 2005-2006 and 2007-2008. ^2 ^Differences were tested using Student's t test. Comparing with 1989: * P < 0.05, comparing with 2005: † P < 0.05.

Three major beverage groups of SSBs showed high per capita increases and also significant increases in the percent consuming from 1989 to 2008. In terms of percent consuming, fruit drinks and soft drinks increased from 67% to 77%; high fat high sugar milk increased from 19% to 39% (P < 0.05), and sports drinks increased from 2% to 12% (P < 0.05) (Figure 2). Parallel increases in kcal/d per consumer occurred as well. Significant short-term trends, from 2005 to 2008, included a significant increase in high fat high sugar milk (28% to 39%, P < 0.05).

### Trends in milk consumption

**Figure 3:**
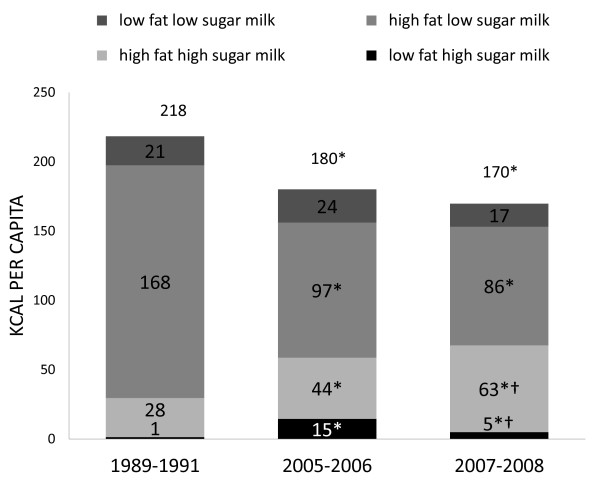
**Total kcal per capita from milk groups in US children 6-11 y old 1989-2008, nationally representative^1, 2^**. ^1 ^The studied sample included children aged 6-11 y old from 3 nationally representative surveys: CSFII 1989-1991, NHANES 2005-2006 and 2007-2008. ^2 ^Differences were tested using Student's t test. Comparing with 1989: * P < 0.05, comparing with 2005: † P < 0.05.

Trends in milk consumption shifted from 1989 to 2005-2008 (Figure 3). Total per capita intake of milk decreased over this time period, from 218 to 170 kcal/d, P < 0.05. The four milk categories vary in saturated fat and sugar content. Caloric milks are those high in saturated fat and added sugar, and include high fat high sugar, high fat low sugar, and low fat high sugar milks. Consumption of high fat low sugar milk by this age group declined significantly (168 to 86 kcal/d, P < 0.05), though high fat high sugar milk intake increased substantially over the same time period (28 to 63 kcal/d, P < 0.05).

### Trends in low calorie beverage consumption

**Figure 4:**
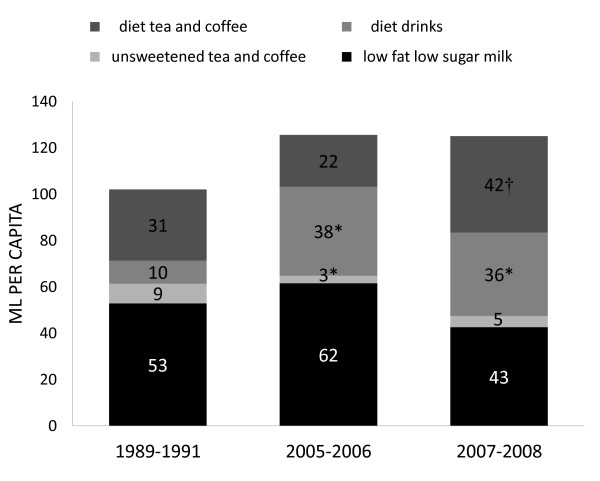
**Total mL per capita from healthier beverages in US children 6-11 y old, 1989-2008, nationally representative^1, 2^**. ^1 ^The studied sample included children aged 6-11 y old from 3 nationally representative surveys: CSFII 1989-1991, NHANES 2005-2006 and 2007-2008. ^2 ^Differences were tested using Student's t test. Comparing with 1989: * P < 0.05, comparing with 2005: † P < 0.05.

Per capita calories from low calorie beverages remained significantly consistent from 1989 to 2008 among children aged 6-11 (Figure 4). Beverages in this category include low fat low sugar milk, unsweetened tea and coffee, and diet tea, coffee, and soft drinks. Over this time period, the decrease in mL/d of low fat low sugar milk was not significant, however, a significant increase in mL/d for all diet drinks occurred (diet drinks increased from 10 to 36 mL/d from 1989-2008, P < 0.05 value, and diet tea and coffee from 31 to 42 mL/d from 2005-2008, P < 0.05) (Table 3).

### Differential trends in SSB and juice consumption among ethnic groups

Noteworthy differences in trends among ethnicities were found (Table 4). Per capita consumption of fruit drinks and soft drinks increased most significantly among non-Hispanic Blacks (79 to 134 kcal/d, P < 0.05), followed by Hispanics (71 to 111 kcal/d, P < 0.05) and non-Hispanic Whites (92 to 121 kcal/d, P < 0.05). Non-Hispanic Whites had increased per capita intake of high fat high sugar milk (28 to 63 kcal/d, P < 0.05), followed by Hispanics (39 to 75 kcal/d, P < 0.05). Non-Hispanic Blacks were the only group to show a significant per capita increase for 100% fruit juice (31 to 62 kcal/d, P < 0.05).

**Table 4:** Per capita trends (Kcal/day), % Consumers, Amount per consumer (Kcal/day). Children aged 6-11 y old by Ethnicity. US Nationally Representative, 1989-2008^1, 2^.

	CSFII 1989-1991					NHANES 2005-2006					NHANES 2007-2008				
	Per Capita Trends (kcal/day)		**% Cons**.	Amt. Per Consumer (kcal/day)		Per Capita Trends (kcal/day)		**% Cons**.	Amt. Per Consumer (kcal/day)		Per Capita Trends (kcal/day)		**% Cons**.	Amt. Per Consumer (kcal/day)	
**Hispanic**															
fruit drinks & soda	71	± 15	64%	110	± 19	119*	± 6	88%	134	± 5	111*	± 9	77%	144	± 9
high fat high sugar milk	39	± 16	20%	199	± 46	60	± 9	38%*	159	± 12	75*	± 7	47%*	160	± 6
100% juice (fruit + vegetable)	69	± 17	60%	116	± 11	52	± 5	59%	89	± 5	61	± 4	62%	99	± 7
**Non-Hispanic White**															
fruit drinks & soda	92	± 7	66%	140	± 10	110	± 10	74%	148	± 10	121*	± 11	77%	156	± 12
high fat high sugar milk	28	± 6	19%	152	± 12	39	± 7	24%	162	± 12	63*†	± 8	38%*†	166	± 14
100% juice (fruit + vegetable)	41	± 2	45%	90	± 4	37	± 3	43%	85	± 4	41	± 7	44%	94	± 11
**Non-Hispanic Black**															
fruit drinks & soda	79	± 10	68%	117	± 9	131*	± 6	87%	151	± 7	134*	± 7	84%	161	± 6
high fat high sugar milk	21	± 7	19%	111	± 9	51	± 8	32%	156	± 15	49	± 5	32%	153	± 6
100% juice (fruit + vegetable)	31	± 5	39%	79	± 10	64*	± 6	64%*	99	± 6	62*	± 5	59%*	105	± 5

^1 ^The studied sample included children aged 6-11 y old from 3 nationally representative surveys: CSFII 1989-1991, NHANES 2005-2006 and 2007-2008.

^2 ^Differences were tested using Student's t test. Comparing with 1989: * P < 0.05, comparing with 2005: † P < 0.05.

## Discussion

Our results offer new insight into broad trends as well as individual beverage trends. With a steady per capita kcal contribution from total beverages over the study period, per capita consumption of SSBs increased while that of CNBs decreased. The substantial increases in the consumption of certain SSBs, such as fruit drinks and soda, high fat high sugar milk, and sports drinks, coupled with the decreases in consumption of high fat low sugar milk were driving forces for this shift. Increases in the percent consuming SSBs, as well as in the amount per consumer are reason for concern, as the childhood obesity prevalence parallels these trends. Increased caloric contribution from high fat high sugar milk signifies that sweetened milk is one of the major SSBs currently marketed towards this age group. Important differences in consumption trends of certain SSBs and 100% juice among ethnicities suggest that determining strategies to reduce caloric beverage intake for school-aged children is complex.

Many of our findings were consistent with those of previous studies. Despite recent efforts to reduce SSBs in schools, SSB per capita trends continued to increase for 6-11 y olds [4,8]. One previous study (1977-1998) found increased consumption of both regular and diet soda as well [9,12].

Most noteworthy is the increased consumption of high fat high sugar milk. Our definition of "high fat" followed the current 2010 Dietary Guidelines for Americans that promote intake of skim or low fat (1%) milk and also the cut-off point being used by the USDA to determine whether milk is low fat or not for school milk programs. This increasing pattern was also showed across many nations in Europe during this same 2007-8 period [13]. Duffey et al. report for adolescents across nine European countries high consumption of high fat high sugar milk [13]. This suggests that a worldwide promotion of the beverage companies to shift children and adolescents toward high sugar and high fat milk may be a key element of their marketing strategy.

Consistent with other studies, our results indicated a decline in total milk consumption, which is a cause for concern for this age group [10,12,28]. SSBs other than sweetened milk and diet drinks that showed significantly large increases in per capita trends over the study period are likely displacing milk. A previous study found evidence for milk being displaced by diet soda [10]. Along with two other studies, it also showed evidence supporting the displacement of milk by SSBs [11].

A study from 2008 reported increased per capita consumption of 100% fruit juice for 6-11 y olds from 1988-1994 to 1999-2004 (34 to 41 kcal/d, P < 0.05) [8]. Our results indicated no significant increase in crude per capita consumption trends of 100% juice, however, non-Hispanic blacks showed a significant increase.

### Limitations

Inherent limitations exist in using surveys that differ in methodology, in this case, the USDA CSFII89 survey and the NHANES continuous surveys. No bridging studies have been carried out to determine whether methodological changes have caused systematic changes in reporting [29,30]. The food and beverage grouping system used by our research group was designed to provide consistency in nutrient value estimates over time [2].

Dietary intake recall surveys are subject to recall bias and underreporting. In addition, our data is predominantly based on recall by a proxy, such as a parent or other caregiver. Two days of dietary intake information may not reflect usual dietary intake. Underreporting has been shown to increase with age for children, and is commonly associated with the reporting of unhealthy foods [31,32]. Additionally, differences in gender and ethnicity contribute to differential underreporting [32].

In this study, differential trends for demographic categories including maternal education level, income level, and ethnicity were not reported. These were under-powered due to the sample size not being large enough.

### Implications and Policy Considerations

Our findings suggest that SSBs in addition to fruit drinks and soda, such as sweetened high fat milk and sports drinks, should be targeted in the effort to reduce caloric intake from beverages for 6-11 y olds. It is unclear whether increased consumption of diet drinks, which are considered healthier alternatives, are safe for this age group. Most of these beverages contain caffeine, which may adversely affect the development of the nervous system at high doses [33].

These results indicate that further interventions to reduce SSB consumption within this age group should be considered. Recent efforts have been made to limit SSB consumption at schools, including the efforts made by the American Beverage Association with the support of the Clinton Foundation and the American Heart Association. We showed important trends in caloric beverage consumption in the period before and during the implementation of this initiative. However, another recent study found that for US children ages 2 to 18, the majority of SSBs were consumed in the home (55% to 70% depending on age), compared to a small percentage consumed at school (1% to 5%) [8]. The taxing of SSBs has been proposed to reduce overall intake of SSBs and influence healthier beverage purchases, which would influence children's consumption of these beverages at home [34].

## Conclusion

Our findings of increased caloric contribution from SSBs, especially high fat high sugar milk, soft drinks and sports drinks, are a cause for concern as they mirror the current trends of childhood obesity. A decline in total milk consumption adds to the nutritional implications for this age group. Current trends suggest the need for policy initiatives targeting children's consumption of other SSBs in addition to fruit drinks and soda.

## Abbreviations

CNB: Caloric Nutritional Beverages; CSFII: Continuing Survey of Food Intake by Individuals; LCB: Low Calorie Beverages; NHANES: National Health and Nutrition Examination Survey; SSB: Sugar-Sweetened Beverages; USDA: U.S. Department of Agriculture.

## Competing interests

The authors declare that they have no competing interests.

## Authors' contributions

GL, CP and BMP had full access to all study data and take full responsibility for the integrity of the data and accuracy of the analysis. All authors read and approved the final manuscript.

BMP conceptualized and designed the study, critically revised the manuscript for important intellectual content, obtained funding, and supervised the study. GL, CP and BMP analyzed and interpreted the study. GL, CP and BMP drafted the manuscript and provided statistical expertise, administrative, technical, and material support.
